# Coakley on the Nose and Throat

**Published:** 1899-10

**Authors:** 


### BOOK REVIEWS, PAMPHLETS, EXCHANGES.

---

### BOOK REVIEWS.

[Contributions solicited for review.]

---

COAKLEY ON THE NOSE AND THROAT. The Diagnosis and Treatment of Diseases of the Nose, Throat, Nasopharynx and Trachea. For the use of Students and Practitioners. By Cornelius G. Coakley, M.D., Professor of Laryngology in the University and Bellevue Hospital Medical College, New York. In one handsome 12mo volume of 536 pages, with 92 engravings and 2 colored plates. Cloth, $2.75, *net*. Lea Brothers & Co., Philadelphia and New York.

Another work on the Nose and Throat has been presented to the medical profession. They seem to come " thick as autumnal leaves that strew the vale of Valambrosa." Dr. Coakley's book, like all others, is but a compilation and expression of the author's on views upon the subject. It seems to us that a book in the form of a manual on the nose and throat should also include the ear, for there are few men who treat the former who do not also treat the latter, and the expense of buying two books is ofttimes no small item. The author has given to the profession a very succinct and well-written volume, and which in the main is safe to follow. In such a book, however, it is impossible for the author to treat a subject more than in the merest details. The arrangement and classification of subjects are very good. Some of the author's views do not agree with those of our own, but this of course is immaterial. For instance, throughout the book we find the author using such expressions as "spray the throat" or "spray the nose with 2 per cent. solution of cocaine." This procedure we do not think should ever be advised, because it is unnecessary and likely to lead to bad results. We believe that whatever portion of the nose or throat is to be treated when cocaine is needed should be touched with this solution on a cotton swab, and therefore confined to a very narrow area. All of the toxic effects from cocaine which we have ever seen have come from spraying cocaine. In atrophic rhinitis the author fails to mention as one of the treatments the use of cupric electrolysis, which is now recognized as of great value. However, it is much easier to criticize than to write a book. The author is to be congratulated and the publishers commended for the general excellency of the work. R.

